# Forty-Nine Cases of Artificial Dentures Swallowed

**Published:** 1905-06-15

**Authors:** Arthur D. Black


### FORTY-NINE CASES OF ARTIFICIAL DENTURES SWALLOWED.

ARTHUR D. BLACK, B.S., M.D., D.D.S.

In making a classified card index of the articles published in the *Dental News Letter* and *Dental Cosmos* a few years ago, many interesting and often perplexing questions came up as to where certain articles would be best classified. Many changes were found to be necessary in the classification made out at the beginning, and quite a number of entirely new divisions were required. It is doubtful if any one, in making out a list of classes into which articles published in our dental literature might be conveniently grouped, would think a division necessary for those on artificial dentures swallowed, yet such proved to be the case. The first few of the articles were classified under artificial dentures, but as the work progressed, the number gradually increased until there were enough to justify placing them in a separate group. Out of about twenty thousand articles classified, extending over a period of fifty-six years, there were reports of forty-nine cases of artificial dentures swallowed.

I have gone over these reports very carefully and have tabulated them in various ways with a view of bringing out some of the interesting facts. It is rather wonderful to notice nature's tolerance in some of the cases, and the apparent lack of sensation manifested by several patients who swallowed plates and retained them in the esophagus for a considerable time, several months in two cases, without the slightest suspicion that there was any particular trouble —only a sore throat. It is rather wonderful, too, that some of these plates, carrying from six to ten teeth, could have been swallowed at all, and when we notice that plates carrying five, six and even seven teeth have passed entirely through the digestive tract without any injurious effects or much discomfort, it helps us to realize that we are really wonderfully made. It is also interesting to note that six cases were operated upon, three esophagotomy, two laryngotomy and one tracheotomy, and all recovered. Several of these operations were performed nearly fifty years ago.

Of the forty-nine plates swallowed, thirty-four were metal, eight of vulcanite, seven material not mentioned.

The number of teeth on the various plates was as follows: One without any, five had one each, ten had two each, four had three each, six had four each, five had five each, three had six each, two had seven each, one had eight, one had nine, one had ten, ten number of teeth not mentioned. Many of them had clasps, which were, probably, the most dangerous parts, and were doubtless the cause of several of the deaths. I think it likely that most of these plates were of the "horse-shoe" variety, carrying one or more of the upper front teeth. This style of plate was a very common one, the custom among dentists for many years being to make either a full denture or a "horse-shoe" carrying "a set of fours," or a "set of six" front teeth. This might account for the cases in which the plates remained in the esophagus so long without discomfort, the palatal portion of the plate fitting closely to the wall of the esophagus and not offering much obstruction to the passage of food.

The conditions under which the plates were swallowed were the following: Seventeen during sleep, ten during fits, mostly epileptic, one during puerpural convulsions, seven while eating or drinking, one during a fall, thirteen condition not mentioned.

Of the forty-nine plates, nine lodged in the pharynx, twenty-seven in the esophagus, two in the trachea and one in the stomach. Nine passed through the entire digestive tract without giving the patients any trouble, although several of these must have lodged somewhere in the stomach or intestines as shown by the time occupied in passing through. One was not found, although the patient died and a post-mortem was held. Three of those lodging in the esophagus were pushed through into the stomach and passed on without difficulty, making a total of twelve that passed through the bowels. Of these three had two teeth, one had three, one had four, two had five, two had six, one had seven, two number of teeth not mentioned.

One of the most interesting tabulations is that of the number of days occupied in that passage of these plates. One was two days, two were three days, one was five days, two were seven days, one was nine days, one was twelve days, one was one hundred and eleven days, and three time not mentioned. It is rather remarkable that the plate which was in the body one hunderd and eleven days did not cause any serious trouble. We would think that there would have been an erosion of the tissues where it was lodged, or that it would have caused an obstruction.

Of the nine plates lodged in the pharynx, one caused immediate death by lodging above the epiglottis and closing it. Another remained in the pharynx four and one-half months and was removed post-mortem, the patient dying of bronchitis. Five were removed through the mouth, by the finger or forceps, after remaining in the pharynx varied periods of from a few hours to five months. The plate that remained in for five months was a rubber piece carrying five teeth, swallowed during sleep. The patient had complained of sore throat, but never suspected having swallowed the plate. The other two were removed by pharyngotomy and recovered. A peculiar result of one of these operations was the changing of the patient's voice from tenor to bass, supposedly on account of the vocal cords being thickened afterward.

Of the twenty-seven lodged in the esophagus, two caused ulceration into the aorta and death, one was pushed down the esophagus nearly to the stomach and remained there three months, when death intervened. Three were pushed into the stomach and passed through the bowels. One remained in the esophagus nineteen days, was pushed into the stomach and was vomited ninety-seven days later, making a total of one hundred and sixteen days in the body. Five others were either vomited or coughed up, one of the patients dying eight days later as a result of inflammation. Another remained in the esophagus fifteen months and the patient, who had swallowed it during a fit, never suspected it to be the cause of a sore throat during all these months. This plate was coughed up. Ten of this group were fished out via the mouth, having remained in from a few hours to three and one-half months. Three were removed by operation of esophagotomy and all three patients recovered.

Of the two in the trachea, one caused death in a short time, the other was removed by operation and the patient recovered.

The one mentioned as having lodged in the stomach was fished out with an esophageal coin catcher.

One plate carrying two teeth was swallowed and the patient died a week later. The plate could not be found post-mortem.

There were ten fatalities out of the forty-nine cases, as follows: Four caused ulceration through wall of esophagus into aorta, pericardium or lung. One died of bronchitis, one on account of closure of the glottis, one lodged in the trachea, causing asphyxiation, one was found low in esophagus, post-mortem, death having occurred in three months; one died of inflammation after having vomited plate up, and one died a week after swallowing plate, but plate could not be found post-mortem.

Thirty-nine patients recovered, of these cases, twelve plates passed through the entire digestive tract, fifteen were fished out through the mouth, six were vomited or coughed up, and six were removed by operations through the neck.—*Northwestern Journal.*

